# Association of FV G1691A, FV H1299R, and FII G20210A variations with thrombosis and coronary artery disease (CAD): A population-based study

**DOI:** 10.5937/jomb0-39668

**Published:** 2025-06-13

**Authors:** Özmen Sevda Ünallı, Yeşim Özarda, Aylin Köseler, Ramazan Sabırlı, Derya Sucu Kaynak, Ibrahim Koç

**Affiliations:** 1 City Hospıtal, Central Laboratory, Department of Medical Biochemistry Bursa, Turkey; 2 Uluda University, Health Sciences Institute, Department of Translational Medicine, Bursa, Turkey; 3 Yeditepe Unıversity, Faculty of Medicine, Department of Medical Biochemistry, Istanbul, Turkey; 4 Pamukkale University, Faculty of Medicine, Department of Biophysics, Denizli, Turkey; 5 Servergazi State Hospital, Department of Emergency Medicine, Denizli, Turkey; 6 Head Office, Department of Occupational Health and Safety Specialist, Bursa, Turkey; 7 City Hospıtal, Department of Pulmonary Medicine Clinic, Bursa, Turkey

**Keywords:** thrombosis, coronary artery disease, factor V gene, prothrombin gene, gene variations, allele frequencies, tromboza, bolest koronarnih arterija, gen za faktor V, gen za protrombin, varijacije gena, frekvencija alela

## Abstract

**Background:**

Thrombosis and coronary artery disease (CAD) are complex disorders influenced by genetic factors. Specific gene variations, such as Factor V (FV) G1691A (Leiden), FV H1299R, and Prothrombin (FII) G20210A, have been implicated in thrombotic events and CAD. However, their precise role in CAD development remains controversial. This study investigated the prevalence and association of these gene variations with thrombosis and CAD in the Turkish population.

**Methods:**

A case-control study included 406 healthy individuals and 64 CAD patients. Genotyping for FV G1691A, FV H1299R, and FII G20210A was performed using a strip assay. Fisher's exact test compared allele and genotype frequencies between the CAD and control groups.

**Results:**

No significant differences were observed in genotype frequencies of FV G1691A, FV H1299R, and FII G20210A between the CAD and control groups (p>0.05). Similarly, allele frequencies did not differ significantly between the two groups (p>0.05).

**Conclusions:**

The findings suggest that FV G1691A, FV H1299R, and FII G20210A variations may not play a significant role in the development of CAD in the Turkish population studied. These results are consistent with the existing conflicting literature on the association between these gene variations and CAD. Further research with larger sample sizes and diverse populations is warranted to elucidate the role of these variations in CAD pathogenesis.

## Abstract (sr)

**Uvod:**

Tromboza i bolest koronarnih arterija (CAD) su složeni poremećaji na koje utiču genetski faktori. Specifične genetske varijacije, kao što su Faktor V (FV) G1691A (Leiden), FV H1299R i protrombin (FII) G20210A, su povezane sa trombotskim događajima i CAD-om. Međutim, njihova tačna uloga u razvoju CAD-a ostaje nejasna. Ova studija je istraživala prevalenciju i povezanost ovih genetskih varijacija sa trombozom i CAD-om u turskoj populaciji.

**Metode:**

Studija slučaj-kontrola je obuhvatila 406 zdravih osoba i 64 pacijenta sa CAD-om. Genotipizacija za FV G1691A, FV H1299R i FII G20210A je izvedena korišćenjem strip testa. Fišerov egzaktni test je korišćen za poređenje učestalosti alela i genotipova između grupe sa CAD-om i kontrolne grupe.

**Rezultati:**

Nisu uočene značajne razlike u učestalosti genotipova FV G1691A, FV H1299R i FII G20210A između grupe sa CAD-om i kontrolne grupe (p>0,05). Slično tome, učestalost alela takođe nije pokazala značajne razlike između ove dve grupe (p>0,05).

**Zaključak:**

Rezultati ukazuju na to da varijacije FV G1691A, FV H1299R i FII G20210A možda nemaju značajnu ulogu u razvoju CAD-a kod ispitanika u turskoj populaciji. Ovi nalazi su u skladu sa postojećom kontradiktornom literaturom o povezanosti ovih genetskih varijacija i CAD-a. Neophodna su dalja istraživanja na većim uzorcima i raznovrsnijim populacijama kako bi se razjasnila uloga ovih varijacija u patogenezi CAD-a.

## Introduction

Thrombosis poses a significant global health challenge due to its high rates of mortality and morbidity. The development of thrombosis involves various factors, including environmental, clinical, and genetic influences. Similarly, the development of coronary artery disease (CAD) entails a multifaceted aetiology, primarily encompassing a combination of traditional risk factors such as type 2 diabetes, dyslipidemia, arterial hypertension, cigarette smoking, as well as genetic predisposition. Factor V (FV) and Prothrombin (FII) gene variations are commonly associated with thrombotic events. Still, the role of specific variants, such as factor V Leiden (FVL), prothrombin gene (PT G20210A), and methylenetetrahydrofolate reductase (MTHFR) C677T polymorphisms in coronary artery disease (CAD) development remains controversial.

### Role of Factor V gene variations in thrombotic events

Familial thrombophilia, a common cause of inherited thrombosis, is often associated with Activated Protein C Resistance (APCR). APCR is primarily caused by a single variation in the Factor V (FV) gene, known as Factor V Leiden (FVL), located on chromosome 1. The FVL mutation is inherited in an autosomal dominant manner. This variation involves the substitution of guanine with adenine at nucleotide 1691 in the FV gene, replacing arginine with glutamine at the 506th amino acid position (Arg506Gln) of the FV protein. This variation occurs at the site of the first molecular cleavage of factor Va by Activated Protein C (APC). Due to this variation, thrombin is continued to form by the prothrombinase complex [1] [2] [3] [4].

The number of FVL mutant alleles influences the clinical manifestation of FVL mutation-related thrombophilia. Certain circumstantial risk factors, such as central venous catheters, organ transplantation, contraceptive use, advancing age, hormone replacement therapy, surgery, and prolonged immobilization, such as in travel, can have an additive effect on thrombotic events (5). Heterozygote genotype frequencies of FVL variation range from 3% to 8% in the United States and Europe general populations. These frequencies vary significantly, with higher frequencies observed in European populations and lower frequencies in African, indigenous Australian, and Asian populations. The homozygous genotype frequency of the FVL variation is approximately 1 in 5000 individuals [5].

In addition to the FVL variation, sequencing of exon 13 of the FV gene has revealed a 4070A-G variation (His1299Arg; H1299R), known as the HR2 haplotype. This variation is associated with several other polymorphisms [6]. The HR2 haplotype encompasses more than 12 different polymorphisms throughout the FV gene (collectively known as HR2), with seven polymorphisms predicting amino acid changes in the FV protein and resulting in protein modifications [7] [8]. Carriership of the FV H1299R allele is associated with mild APCR and interacts with the FVL variation, leading to a more severe APCR phenotype [9] [10] [11]. However, the specific thrombotic event risk associated with the HR2 haplotype remains unclear [3] [12]. Nevertheless, the presence of both the FV H1299R allele and the FVL variation has increased the risk of thrombotic events even further [9] [13].

### Role of prothrombin gene variation in thrombotic events

Following the Factor V Leiden (FVL) variation, the prothrombin or FII G20210A variation is the second most common genetic risk factor associated with thrombosis [14] [15] [16]. The G20210A mutation is found in approximately 1–4% of healthy individuals, and its prevalence varies among populations. The highest prevalence is observed in Europe, while G20210A heterozygosity is extremely rare in Asian, African, and Native American populations. The prevalence of G20210A homozygosity is approximately one in 10,000 individuals [17] [18] [19].

The FII G20210A variation is a genetic polymorphism located in the 3’-untranslated region of the prothrombin (FII) gene. It is caused by a single base pair substitution, where guanine (G) is replaced by adenine (A) at position 20210. The inheritance of this variation is autosomal dominant and is associated with elevated plasma levels of prothrombin (FII) [17]. Additionally, the FII G20210A variation has been implicated in the risk of arterial disease. Young women with the FII 20210A allele were reported to have a fourfold higher risk of myocardial infarction, while men had a 1.5-fold higher risk [18].

Adult individuals carrying the G20210A heterozygote genotype have a two- to fourfold increased risk of thrombosis, while children with this genotype face an even higher three- to fourfold increased risk. Furthermore, individuals with G20210A homozygosity tend to experience thrombotic events more frequently and at a younger age. The FII variation has also been associated with an increased risk of preeclampsia and pregnancy loss [19] [20].

This case-control and prevalence study aims to determine the allele frequencies of the FV gene G1691A-H1299R and FII gene G20210A mutations as genetic risk factors for thrombosis and coronary artery disease in both the healthy population and patients with coronary artery disease in the South Marmara region of Western Turkey.

## Materials and methods

### Ethical issues, study population, and blood sampling

Ethical approval for the study was obtained from the ethics committee of Uluda University, ensuring compliance with ethical guidelines. All participants were provided with information about the study and their rights, and their informed consent was obtained following the principles outlined in the Helsinki Declaration.

The study population consisted of 406 healthy volunteers (179 males and 227 females) between the ages of 18 and 45 years from the Bursa region. These individuals were included to determine the genotype distributions of the Factor V (FV) and Prothrombin (FII) polymorphisms in the healthy population. In addition, a group of 64 patients (12 females and 52 males) aged between 18 and 45 years with documented coronary artery disease (CAD), confirmed by coronary artery angiography, were included in the study. For all participants, blood samples were collected using EDTA tubes (Vacutainer, Becton Dickinson, U.K.).

### Genotyping

Genotyping for cardiovascular disease (CVD) in this study was performed using the CVD-StripAssay (ViennaLab Labordiagnostika GmbH, Austria). This assay is based on the reverse hybridization principle for mutation analysis, allowing for the simultaneous detection of Factor V (FV) G1691A-H1299R and Prothrombin (FII) G20210A mutations.

Variation analyses were conducted following the manufacturer’s recommendations. DNA was extracted from anticoagulated blood using the Invisorb® Spin Blood Mini Extraction Kit (Invitek, Germany) and stored at -20°C for later use. Subsequently, relevant gene sequences were amplified and biotin-labelled in a single amplification reaction using the PCR amplification method.

In brief, PCR amplification was carried out in a final volume of 25 mL, consisting of 15 mL of pre-prepared PCR amplification mix, 4.8 mL of diluted buffer, 1U of Taq DNA Polymerase (Fermentas), and 5 mL of DNA. The thermal cycler (Applied Biosystems 2720 Thermal Cycler, USA) program included an initial step of 94°C for 2 minutes, followed by 35 cycles of 94°C for 15 seconds, 58°C for 30 seconds, 72°C for 30 seconds, and a final extension step of 72°C for 3 minutes.

Following amplification, the products were selectively hybridized using an Autolipa automated incubator (ProfiBlot T48, TECAN, Switzerland) on a test strip that contained immobilized allele-specific (wild-type and mutant) oligonucleotide probes arranged as an array of parallel lines. Biotinylated sequences bound to the strip were detected using streptavidin-alkaline phosphatase and colour substrates. The genotype of each sample was determined by comparing the staining pattern of the processed Test strip with the Decoder table enclosed in the assay kit. Each polymorphic position displayed one of the following staining patterns: Normal, Heterozygous, or Homozygous mutant [21].

Genotyping for cardiovascular disease (CVD) was performed using the CVD-StripAssay based on the reverse hybridization principle. DNA was extracted from anticoagulated blood, and PCR amplification was conducted following the manufacturer’s recommendations. The genotype of each sample was determined by comparing the staining pattern on the Test strip with the Decoder table provided in the assay kit.

### Statistical analysis

The frequency of Factor V (FV) Gene G1691A (Leiden)-FV Gene H1299R and Prothrombin (FII) Gene G20210A genotypes and alleles were analyzed using the Statistical Package for the Social Sciences program (SPSS for Windows, version 17.0; SPSS, Chicago, IL).

Fisher’s exact test was employed to compare allele and genotype frequencies between the two groups. A p-value of less than 0.05 was considered statistically significant for all analyses, indicating a significant difference between the compared groups.

## Results

The findings regarding the genotype frequencies of FV G1691A are presented in Table 1. The results indicate no significant difference between the coronary artery disease (CAD) and control groups regarding FV G1691A genotype frequencies (p=0.170). Similarly, Table 1 displays the FV Gene H1299R genotype frequencies, revealing no significant difference between the control and CAD groups (p=0.544).

**Table 1:** Distributions of mutations in healthy individuals and CAD Group. OR=Odds Ratio; p-values are derived from the Fisher Exact Test; N/E=Not estimated; CI: Confidence Interval

		Control Group <br>n=406	CAD Group <br>n=64	OR (95% CI <br>for Genotypes)	p-value
Polymorphisms	Genotypes	n (%)	n (%)		
Factor V <br>G1691A(Leiden)	AA	1 (0.25)	1 (1.56)	1.45 (0.70–2.89)	0.170
	GA	34 (8.37)	7 (10.93)		
	GG	371 (91.38)	56 (87.5)	0.95 (0.86–1.05)	
Factor V Gene <br>H1299R	GG	2 (0.49)	0	1.32 (0.68–2.59)	0.544
	AG	41 (10.10)	9 (14.07)		
	AA	363 (89.41)	55 (85.93)	0.96 (0.86–1.06)	
Prothrombin <br>G20210A	AA	0 (0)	0	N/E	0.500
	GA	19 (4.68)	1 (1.56)	0.33 (0.04–2.45)	
	GG	387 (95.32)	63 (98.44)	1.33 (0.99–1.07)	

Table 1 also presents the genotype frequencies of FII G20210A. The results indicate no statistically significant difference between the groups regarding FII G20210A genotype frequencies (p=0.500). Table 2 shows the allele frequencies for the FV G1691A, FII G20210A, and FV Gene H1299R polymorphisms. No statistically significant difference was found in allele frequencies between the patient and control groups for all three gene polymorphisms (p>0.05).

**Table 2:** Distributions of Alleles in healthy individuals and CAD group. OR=Odds Ratio; p-values are derived from the Fisher Exact Test; CI: Confidence Interval

		Control Group <br>n=812	CAD Group <br>n=128	OR (95% CI <br>for Alleles)	*p*-value
Polymorphisms	Alleles	n (%)	n (%)		
Factor V G1691A	G	776 (95.56)	119 (92.96)	0.97 (0.92–1.23)	0.187
	A	36 (4.44)	9 (7.04)	1.58 (0.78–3.21)	
Factor V Gene <br>H1299R	A	767 (94.45)	119 (92.96)	0.98 (0.93–1.03)	0.538
	G	45 (5.55)	9 (7.04)	1.26 (0.63–2.53)	
Prothrombin <br>G20210A	G	793 (97.66)	127 (99.21)	1.01 (0.99-1.03)	0.504
	A	19 (2.34)	1 (0.79)	0.33 (0.45–2.47)	

## Discussion

Our study investigated the genotype and allele frequencies of FV G1691A (Leiden), FV H1299R, and FII G20210A variations concerning coronary artery disease (CAD) in the Turkish population. The results showed no significant differences between these mutations’ genotype and allele frequencies in the CAD and control groups. This suggests that these gene polymorphisms may not play a significant role in the development of CAD in the studied population.

Our findings are consistent with previous studies that have reported conflicting results regarding the association between these gene polymorphisms and CAD. Some studies have reported an increased risk of CAD associated with these variations, while others have found no significant association.

The prevalence of FV G1691A (Leiden) and FV H1299R variations in our study aligns with findings from studies on Turkish and Greek populations. Still, it differs from the European and Asian populations, suggesting genetic variations among different populations [22] [23] [24] [25] [26] [27] [28] [29] [30] [31]. These discrepancies can be attributed to genetic variations among different populations.

Similarly, the association between FII G20210A variation and CAD has yielded conflicting results in previous studies. Some studies have suggested an increased risk [32] [33] [34] [35] [36], while others have found no significant association [37] [38]. Our study did not find a significant difference in the genotype and allele frequencies of FII G20210A between the CAD and control groups.

In conclusion, our study found no significant association between FV G1691A (Leiden), FV Gene H1299R, and FII G20210A variations and CAD in the studied Turkish population. The prevalence of these variations aligned with studies on Turkish and Greek populations but differed from the European population. Further research with larger sample sizes and diverse populations is needed to understand these variations’ role in CAD development comprehensively.

## Dodatak

### Conflict of interest statement

All the authors declare that they have no conflict of interest in this work.
